# The Health Benefits of the Bioactive Compounds in Foods

**DOI:** 10.3390/foods10020325

**Published:** 2021-02-04

**Authors:** Laura Jaime, Susana Santoyo

**Affiliations:** Institute of Food Science Research (CIAL), Universidad Autónoma de Madrid (CEI, UAM-CSIC), 28049 Madrid, Spain

**Keywords:** bioactive compounds, biological activities, isolation, analysis, mechanism of action, bioaccessibility, intestinal absorption, bioavailability

The health benefits of consuming certain foods have been commonly known since ancient times. However, the study of foods as a source of healthy bioactive compounds has been gaining interest over recent decades. At present, numerous research papers have been focused on the beneficial role played by certain food components in the close relationship between food intake and health status. In this sense, many foods, including fruits, vegetables, fish, seaweeds, herbs, etc., are known to be excellent sources of bioactive compounds such as carotenoids, phenolic compounds, terpenoids, fatty acids, peptides, and saponins, among others.

On the other hand, the development of new foods or nutraceuticals with health benefits is a current topic today and represents an appealing opportunity for the food and/or pharmaceutical industries. However, this launch of new products should be endorsed by strong scientific support on the health benefits attributable to the intake of these bioactive food ingredients. To this end, an enlightenment about the most suitable source of a specific bioactive compound is required. This study should include the most suitable sources of bioactive compounds, the development of the most sustainable extraction techniques, isolation, and also an accurate analysis of the bioactive compounds by using the most adequate techniques. Moreover, the biological activities of these compounds should be elucidated in vitro, in cells, and also in clinical trials. Studies focusing on changes during storage, the digestion process, intestinal absorption rates, bioaccessibility, bioavailability, biological mechanisms of action, or bioactivity of their metabolites are also required to establish the real contribution of these compounds to the health status.

Within this context, food is usually exposed to various temperatures throughout the food supply chain. Thus, research aimed at changes in bioactive compounds that occur during food storage at different temperatures is a topic of interest. In this context, Lu et al. [1] carried out a pilot study to investigate the changes of carotenoids, flavonoids, and vitamin C in a “Cara-Cara” juice during 16 weeks of storage at 4, 20, 30, and 40 °C. The results indicated that total flavonoids and carotenoids studied showed slight degradation at each temperature, while vitamin C degraded intensively, especially at 40 °C storage. Another work [2] also studied the bioactive compounds, antioxidant activity, and sensory analysis of a cryoconcentrated calafate (*Berberis microphylla*) juice during refrigerated storage. This study indicated that under a refrigerated storage time of 35 days, the cryoconcentrate samples retained bioactive compounds and the antioxidant components naturally presented in calafate juice.

On the other hand, food processing techniques could also be related with the amount of bioactive compounds present in foods. Thus, Janda et al. [3] determined the mineral content, antioxidant activity, and acidity of coffee beverages depending on the brewing technique. Their findings showed that the brewing method had a significant effect on the antioxidant potential, polyphenol content, and redox potential of the beverage obtained. Use of the AeroPress coffee maker was the brewing method that resulted in the highest content of health-promoting compounds in a coffee beverage. Besides, there are other processes such as enzyme treatment and fermentation of cereals that enhance the release of bound bioactive compounds and make them available for bioactivity. Authors of [4] found that enzyme-treated destarched rice samples with subsequent fermentation contained known antihypertensive phenolic compounds and peptides that make these samples a promising material for developing cheap antihypertensive foods.

Moreover, many foods have been described to possess different biological activities beyond their nourishing properties, linked to the presence of some constituents. In this Special Issue, the health benefits of donkey milk, quinoa, bee products, and ginger have been included. In that way, quinoa is known to contain some constituents (saponins or dietary fiber polysaccharides, among others) that exert an anti-obesity activity. Nevertheless, its mechanism of action needs to be ascertained. Teng et al. [5] characterized an anti-obesity polysaccharide from quinoa using gas chromatography coupled to mass spectrometry, the structure of which was confirmed by nuclear magnetic resonance. Moreover, they demonstrated that this fructose- and glucose-based polysaccharide inhibited 3T3-L1 adipocyte differentiation by suppressing specific genes’ expression. In other work [6], *Equus asinus* is presented as a novel and improved alternative to cow’s milk due to its great similarity with human milk and its low allergenic properties. However, the properties of donkey milk are not limited to its provision of valuable nutrients, as a wide range of biological activities such as antioxidant, antimicrobial, anti-tumoral, anti-proliferative and anti-diabetic activities compared to other sources of milk have been described, especially associated to whey protein fraction. Moreover, these promising activities might be used by the food industry for the production of novel foods with healthy properties, contributing to immune system stimulation, regulation of intestinal flora, or prevention of inflammatory-based diseases. Regarding foods based on bee products such as royal jelly, propolis, or bee pollen, they have been used since a long time ago to ameliorate some chronic physical states involving increased muscle weakness. In that respect, Ali and Kunugi [7] reviewed that numerous mechanisms at many different levels could be related to a beneficial effect on sarcopenia, including an improvement of inflammation and oxidative damage, enhancement of satellite stem cell responsiveness and muscle blood supply, or promotion of peripheral neuronal regeneration, without ruling out other promising mechanisms. Mao et al. [8] present an up-to-date review concerning the biological activities of ginger, a commonly used spice, that are mainly related to the presence of phenolic compounds such as gingerol and shogaols. In this sense, there are numerous activities that have been described for this spice, from the most known antioxidant or anti-inflammatory activities to anti-diabetic, anti-obesity, or anti-emetic activities. Moreover, the different mechanisms of action to exert these health benefits have been illustrated in this review.

Currently, the valorization of by-products derived from food processing is considered a hot topic. Thus, distilled spent grain, the main residue of baijiu making, is presented as a potential source of melanoidins with antioxidant and antihypertensive activity [9]. In this context, the sequential extraction of melanoidins using water and NaOH at different extraction conditions from dry distilled spent grain, and the subsequent ultrafiltration of the most enriched fraction, makes up a useful technique to obtain fractions enriched in melanoidins with different molecular weights and activities. Melanoidins of high molecular weight (>100 kDa) showed a high antioxidant activity, whereas low molecular weight (3–10 kDa) melanoidins exhibited a high antihypertensive activity against the angiotensin-converting enzyme. Nieto et al. [10] proposed the use of pressurized liquid extraction with green solvents (ethanol:water mixtures) in order to use grape stems as a source of antioxidant phenolics. They optimized the extraction conditions by using an experimental design along with response surface methodology to obtain an extract with high antioxidant activity and high phenolic content. The results pointed out the presence of 42 phenolic compounds, mainly polymer procyanidins, in the optimal extract, where the antioxidant activity was mainly attributed to the presence of the latter. Therefore, this study valorizes this side stream as a source of natural and antioxidant phenolics as part of a sustainable food system.

On the other hand, the encapsulation of bioactive compounds as a strategy to improve their solubility and preserve their chemical integrity and successful delivery in physiological targets has been proposed recently. In that regard, Taha et al. [11] evaluate the efficacy of different milk proteins as nanocarriers for curcumin. Their results revealed that the developed nanoparticles presented an antimicrobial activity much higher compared to curcumin and the native milk proteins.

Nowadays, the colonic microbiota has emerged as a factor in the relationship between diet and health, since increasing evidence connects imbalances in the gut microbiota (dysbiosis) with pathologies. Consequently, there is growing interest in exploring the potential modifications of the gut microbiota by bioactive compounds in foods and their relationship with some pathologies. The study carried out by Ramos-Romero et al. [12] explored the potential modifications in the microbiota profile induced by grape pomace (a product rich in both dietary fiber and polyphenols) supplementation. The authors explored the possible ways in which grape pomace supplementation might have differentially induced a reduction in insulin secretion in responder individuals—specifically changes in representative gut bacterial populations. However, the results obtained in this study indicated that the decrease in insulin levels in subjects at cardiometabolic risk upon supplementation appeared not to be related to modifications in the major subgroups of gut microbiota.

In conclusion, this Special Issue comprises nine original research papers and three review articles addressing recent advances in the health benefits of the bioactive compounds of foods, including new sources of bioactive compounds, valorization of side streams as sources of bioactive compounds, molecular mechanisms of these bioactive compounds, their analysis, isolation, food processing influence on bioactive compounds of food, or the influence of intestinal microbiota, among other related aspects.

## Data Availability

Not applicable.
